# NON-VIRAL REPROGRAMMING OF HUMAN NUCLEUS PULPOSUS CELLS WITH FOXF1 *VIA* EXTRACELLULAR VESICLE DELIVERY: AN *IN VITRO* AND *IN VIVO* STUDY

**DOI:** 10.22203/eCM.v041a07

**Published:** 2021-01-19

**Authors:** S. Tang, A. Salazar-Puerta, J. Richards, S. Khan, J.A. Hoyland, D. Gallego-Perez, B. Walter, N. Higuita-Castro, D. Purmessur

**Affiliations:** 1 Department of Biomedical Engineering, The Ohio State University, Columbus, OH, USA; 2 Department of Orthopedics, The Ohio State University Wexner Medical Center, Columbus, OH, USA; 3 Division of Cell Matrix Biology and Regenerative Medicine, School of Biological Sciences, The University of Manchester, Manchester, UK; 4 NIHR Manchester Musculoskeletal Biomedical Research Centre, Manchester University, NHS Foundation Trust, Manchester Academic Health Science Centre, Manchester, UK

**Keywords:** Intervertebral disc degeneration, low-back pain, cellular reprogramming, engineered extracellular vesicles, transcription factor, electroporation, FOXF1, regeneration

## Abstract

Intervertebral disc (IVD) degeneration is characterized by decreased cellularity and proteoglycan synthesis and increased inflammation, catabolism, and neural/vascular ingrowth. Regenerative methods for IVD degeneration are largely cell-therapy-based or involve viral vectors, which are associated with mutagenesis and undesired immune responses. The present study used bulk electroporation and engineered extracellular vesicles (EVs) to deliver forkhead-box F1 (*FOXF1*) mRNA to degenerate human nucleus pulposus (NP) cells as a minimally invasive therapeutic strategy for IVD regeneration. Bulk electroporation was used to investigate *FOXF1* effects on human NP cells during a 4-week culture in 3D agarose constructs. Engineered EV delivery of *FOXF1* into human IVD cells in monolayer was determined, with subsequent *in vivo* validation in a pilot mouse IVD puncture model. *FOXF1* transfection significantly altered gene expression by upregulating healthy NP markers [FOXF1, keratin 19 (KRT19)], decreasing inflammatory cytokines [interleukin (IL)-1β, −6], catabolic enzymes [metalloproteinase 13 (MMP13)] and nerve growth factor (NGF), with significant increases in glycosaminoglycan accumulation in human NP cells. Engineered EVs loaded with *FOXF1* demonstrated successful encapsulation of *FOXF1* cargo and effective uptake by human NP cells cultured in monolayer. Injection of *FOXF1-*loaded EVs into the mouse IVD *in vivo* resulted in a significant upregulation of *FOXF1* and *Brachyury*, compared to controls at 7 d post-injection, with no evidence of cytotoxicity. This is the first study to demonstrate non-viral delivery of FOXF1 and reprogramming of human NP cells *in vitro* and mouse IVD cells *in vivo*. This strategy represents a non-addictive approach for treating IVD degeneration and associated back pain.

## Editor’s note

The Scientific Editor responsible for this paper was Sibylle Grad.

## Introduction

Chronic LBP is the leading cause of disability worldwide, affecting 70–80 % of the population during their lifetime and many studies have demonstrated IVD degeneration as being a leading cause (Global Burden of Disease Study 2013 Collaborators, 2015; Katz, 2006; Malik *et al.*, 2013; Schwarzer *et al.*, 1995). Furthermore, the economic burden of LBP exceeds $100 billion annually in the USA alone due to the loss of workdays, use of costly interventions, and its role in the growing opioid epidemic (Balagué *et al.*, 2012; Deyo *et al.*, 2015; Katz, 2006). The healthy IVD is avascular and aneural, composed of a core of gelatinous hydrophilic proteoglycans in the central NP encased by concentric collagen I rings that form the AF. The IVD is contained cranially and caudally by the CEPs, which supply nutrients to the disc (Ghosh, 1988). The NP is considered to be the “metabolic engine” of the IVD as it functions to maintain the hydrated core through synthesis of ACAN and COL2, whilst providing load distribution and compressive force absorption (Trout *et al.*, 1982a; 1982b). During IVD degeneration, the NP is characterized by decreased cellularity and proteoglycan synthesis, increased catabolism, along with increases in cell senescence, pro-inflammatory factors, and neural/vascular invasion (Freemont, 2008; Vo *et al.*, 2016). Specifically, studies have demonstrated that degenerated IVDs have decreased ACAN/GAG and COL2 synthesis as well as increased matrix MMPs, proinflammatory cytokines such as IL-1β, IL-6, TNF-α, along with increased NGF (Lama *et al.*, 2018; Le Maitre *et al.*, 2007a; Purmessur *et al.*, 2008; Smith *et al.*, 2011). These changes lead to depressurization of the NP that alters IVD structure and function, resulting in spinal instability.

Many clinical interventions exist for LBP including analgesics, physical therapy, and surgical treatment. However, these interventions only alleviate symptoms while failing to treat the underlying disease pathology (Zhao *et al.*, 2019). Therefore, there is a need for biological interventions that target the early stages of IVD degeneration to reduce/inhibit the degeneration process while also limiting the symptoms of pain. Several potential biological strategies, including the injection of exogenous growth factors (Sobajima *et al.*, 2004), and cell therapies [*e.g.*, MSCs] have been investigated to promote regeneration and reduce inflammation (Loibl *et al.*, 2019; Vadalà *et al.*, 2016). However, several challenges limit their therapeutic potential. For example, growth-factor-based approaches often have a transient therapeutic effect and require repeated dosing. Cell-therapy-based approaches are limited by cell source and long-term efficacy due to poor cell survival in the harsh IVD environment as well as the potential risk for immunogenicity and tumorigenicity (Karagiannis and Yamanaka, 2014; Loibl *et al.*, 2019; Vadalà *et al.*, 2016). As such, cellular reprogramming may be a potential novel therapeutic approach for treatment of IVD degeneration.

Direct cell reprogramming is an alternative biological strategy that has shown promise in other tissues as it addresses many of the limitations of the therapies described above. This approach allows adult somatic cells, which are a widely available cell source, to be transdifferentiated into the desired cell type with the aid of transcription factors, while bypassing the pluripotent state (Karagiannis and Yamanaka, 2014; Takahashi and Yamanaka, 2006). In the present study, reprogramming is defined as reverting somatic diseased cells into a healthy state. Viral vectors are most commonly used for gene delivery due to their simplicity and reproducibility of transfection; however, viral gene delivery has significantly limited clinical application due to the potential for immunogenicity, insertional mutagenesis, and capsid size constraints (Cao *et al.*, 2010; Okita *et al.*, 2008; Sobajima *et al.*, 2004). To circumvent these hurdles, minimally invasive non-viral gene delivery systems that allow direct cell reprogramming through transcription factors are potential approaches that would eliminate much of the potential risks associated with viral-based gene transfer. Transcription factor *FOXF1* has been identified as a potential candidate.

FOXF1 is part of a family of genes involved in the regulation of cell differentiation, growth, and proliferation (Tuteja and Kaestner, 2007). It has recently been identified as a healthy-NP-specific marker with reduced expression during degeneration (Richardson *et al.*, 2017a; Risbud *et al.*, 2015). Deletion of FOX gene clusters in transgenic mouse models leads to spinal and vertebral abnormalities (Stankiewicz *et al.*, 2009). In addition, FOXF1 promotes tissue repair in other organs such as lungs and liver (Bolte *et al.*, 2017; Flood *et al.*, 2019). In a previous study, the developmental transcription factor Brachyury was successful in reprogramming human NP cells from degenerate and painful IVDs to a healthy immature NP-like phenotype (Tang *et al.*, 2019). Thus, FOXF1 could be an additional promising candidate to be explored.

In addition to FOXF1, a mechanism is required for non-viral delivery of the transcription factor into IVD cells in a minimally invasive manner and, thus, the use of engineered EVs is proposed in the present study. EVs are composed of a lipid bilayer comprising transmembrane and cytosolic proteins, DNA fragments, and RNAs that are protected from enzymatic degradation (Valadi *et al.*, 2007). EVs can be classified according to their size, content, and mechanism of generation into multivesicular bodies, exosomes, or apoptotic bodies and play an important role in several processes, including intracellular communication, proliferation, and differentiation (de Jong *et al.*, 2012; Mulcahy *et al.*, 2014). Therefore, they can interact with target cells and release their contents into the cytosol, allowing alteration of gene and protein expression in the recipient cell (Stoorvogel *et al.*, 2002). Consequently, EVs are ideal candidates for gene therapy since they display non-immunogenic characteristics (Ha *et al.*, 2016; O’Brien *et al.*, 2020). Gallego-Perez *et al.* (2017) demonstrated the feasibility of using EVs loaded with a specific transcription factor transcript to achieve direct cell reprogramming of fibroblasts into induced endothelium or induced neurons. These features highlight the potential of engineered EVs to function as a therapeutic delivery system that can replace the use of viral vectors and overcome the caveats associated with viral delivery.

Therefore, the main goal of the present study was to examine the reprogramming potential of non-viral delivery of transcription factors using engineered EVs. The first objective was to examine the effects of *FOXF1* on human NP cells derived from autopsy (mildly-degenerate) and surgery (painful-degenerate) through non-viral bulk transfection with cells cultured in a 3D *in vitro* agarose culture model. The study hypothesis was that *FOXF1* mRNA transcripts could reprogram human NP cells from autopsy and surgery into a healthy phenotype characterized by increases in GAG accumulation, and decreases in inflammatory, catabolic and pain-associated factors. The second objective was to examine the delivery of engineered NP-derived *FOXF1*-loaded EVs to human NP cells in a monolayer culture, with subsequent validation of delivery and *FOXF1* expression in a short-term pilot *in vivo* mouse disc puncture model. The study hypothesis was that human NP cells cultured in monolayer will uptake NP-generated *FOXF1*-loaded EVs and demonstrate increased expression of *FOXF1 in vitro*. Also, these *FOXF1*-loaded EVs could be delivered to the IVD *in vivo* in a mouse IVD injury model with up-regulation of the *foxf1* transcript.

## Materials and Methods

### *In vitro* reprogramming of human NP cells by bulk electroporation of *FOXF1* (Fig. 1)

#### Cell isolation and expansion from human IVD tissue

Lumbar human spines were obtained from autopsy (*n* = 5, 19–58 years old donors) through the CHTN (Institutional IRB exemption) within 24 h post-mortem. IVDs were isolated and graded by three independent investigators according to the Thompson scale and grades were averaged (Table 1: human samples) (Thompson *et al.*, 1990). NP tissue from autopsy was dissected and NP cells isolated as previously described (Tang *et al.*, 2019). Surgical NP tissue from patients undergoing microdiscectomy or lumbar fusion with degenerate IVDs (*n* = 5, 19–60 years old donors), as referenced by magnetic resonance imaging, were obtained from The Ohio State University Wexner Medical Center (IRB:2015H0385). Post dissection, cells were isolated from autopsy and surgical NP tissue using 0.03 g/mL protease (Sigma-Aldrich, Cat: P5147-1G) in digestion medium [DMEM (Corning, Manassas, VA, USA, Cat: 10-013-CV), 4.5 g/mL glucose, 1 % P/S, 0.5 % Fungizone] for 1 h at 37 °C, followed by treatment with 0.03 g/15 mL collagenase II (Thermo Fisher Scientific, Cat: 17101015) for 4 h at 37 °C. The tissue digest was strained through a 70 μm cell strainer to remove cellular debris and cells were plated for expansion (Tang *et al.*, 2019). To assess the effects of *FOXF1* on mild to moderate IVD degeneration, IVDs from autopsy with averaged Thompson grades of 1.5–3 were selected, while surgical cells from patients undergoing surgery for LBP represented the painful IVD degeneration group. Autopsy and surgical NP cells pre-transfection were expanded in disc cell medium (DMEM, 4.5 g/mL glucose, 10 % FBS, 1 % P/S, 0.5 % Fungizone, 50 μg/mL freshly prepared ascorbic acid) in standard culture conditions (5 % CO_2_, 37 °C). Medium was changed every 3 d until 80 % confluency, when the cells were used for downstream transfection (Passage = P2).

#### FOXF1 transcription factor plasmid expansion

A *FOXF1* gene in pCMV6-AC-GFP vector, with antibiotic selection marker ampicillin, was obtained from OriGene Technologies, Rockville, MD, USA (Cat: RG218259, reference sequence from NIH: NM_001451, Human Tagged ORF Clone). Vector pCMV6 plasmids were also obtained and used as sham empty vector controls (Origene, Cat: PS100001) (Table 2: plasmids). As described previously (Tang *et al.*, 2019), *FOXF1* plasmids were transformed into DH5α *Escherichia coli* (*E.coli*) following heat shock and incubated with S.O.C. medium (2 % tryptone, 0.5 % yeast extract, 10 mM NaCl, 2.5 mM KCl, 10 mM MgCl_2_, 10 mM MgSO_4_, 20 mM glucose) for 1 h (37 °C, 225 rpm). Then, bacterial cells were cultured on solid agar (4 % agar in lysogeny broth, Thermo Fisher Scientific, Cat: BP1425-500) for 24 h with ampicillin (100 μg/mL) and selectively cultured in small and large liquid cultures for 24 h. Next, plasmids were isolated from the selectively expanded *E.coli* using a ZymoPure II Plasmid Midiprep Kit (Zymo Research, Irvine, CA, USA, Cat: 4201) per manufacturer protocol and quantified using a Nanodrop 2000c Spectrophotometer.

#### FOXF1 transfection

Human NP cells were expanded to 80 % confluency, as previously described (Tang *et al.*, 2019). Bulk transfection was performed using the Neon Transfection System MPK5000. Previously isolated *FOXF1* and pCMV6 plasmids were mixed with R buffer (0.05 μg DNA/μL) and transfected (1,425 V, 30 ms, 1 pulse) into 1 × 10^6^ cells/transfection, according the following groups (multiple transfections per experimental group): NP cells from autopsy transfected with *FOXF1* (autopsy *FOXF1*), NP cells from surgery transfected with *FOXF1* (surgical *FOXF1*), NP cells transfected with pCMV6 sham vector (autopsy pCMV6, surgical pCMV6). Cells were plated in T-175 cell culture flasks for expansion in antibiotic-free medium (DMEM with 4.5 g/mL glucose, 10 %FBS, 50 μg/mL freshly prepared ascorbic acid) for 48 h before switching to expansion in disc cell medium, as previously described (Tang *et al.*, 2019).

#### 3D agarose culture

Transfected NP cells at ~ 80 % confluency were washed with sterile 1× PBS, trypsinized, and suspended at 40 × 10^6^ cells/mL in disc cell medium (Mauck *et al.*, 2002). As described, a 52 × 32 mm rectangular construct was made using 4 mm-thick sterile silicone sheets (Tang *et al*., 2019). Cells suspended in medium were mixed with equal amounts of 4 % biological grade agarose (Amresco VWR, Solon, OH, USA, Cat: J234) at 50 °C to create a 2 % agarose mixture of 20 × 10^6^ cells/mL. The mixture was quickly homogenized by pipetting, added to the silicone mold sandwiched between two glass plates and allowed to solidify for 10 min at room temperature. Glass plates were removed, and 8 mm diameter sterile biopsy punches were used to create 8 × 4 mm tall cylindrical constructs from the solidified cell-seeded agarose. Individual constructs were cultured in 2 mL of disc cell medium in 24-well cell-repellant culture plates and medium was changed 3 times a week (Tang *et al.*, 2019). Dependent variables including cell viability, gene expression and proteoglycan accumulation were assessed at day 0, week 2, and week 4 post agarose culture.

#### Cell viability

Cell-agarose constructs were divided into two halves, washed with 1× PBS and incubated in 700 μL live/dead solution (Thermo Fisher Scientific, Cat: L3224) for 18 min to stain live and dead cells using green fluorescent calcein-AM (4 mM) and red fluorescent ethidium-homodimer-1 (2 mM), respectively. Then, samples were washed with 1× PBS to remove the residual dye and imaged under fluorescence imaging using a Nikon TiE microscope with a DS-Qi2 camera (Nikon Instruments Inc., Melville, NY, USA). 10× and 4× cross-sectional images were taken of each gel with 10× images used for automatized quantification of cell viability (percentage live cells =s number of live cells over total cells) using MIPAR Image Analysis Software (Tang *et al.*, 2019).

#### Gene expression

mRNA was isolated from the cells seeded in the constructs using the TRIzol Plus RNA Purification Kit (Thermo Fisher Scientific, Cat: 1218355), with the entire cell-agarose gel complex directly digested in TRIzol, and cDNA synthesized using the Maxima H Minus Mastermix (Thermo Fisher Scientific, Cat: M1662) per manufacturer’s protocol (Tang *et al.*, 2019). RT-qPCR was run on 384-well plates with 15 ng of cDNA per reaction using TaqMan Universal Master Mix II (Thermo Fisher Scientific, Cat: 4440049) and TaqMan primers (Table 3). Data were analyzed using the comparative 2^− ΔΔCt^ method normalized to the housekeeping gene *18S* and experimental pCMV6 vector controls (Livak and Schmittgen, 2001).

#### Proteoglycan/GAG content

Constructs were lyophilized and digested in 1 mL of Proteinase K (Roche, Indianapolis, IN, USA, Cat: 03115828001) working solution (1 : 200 Proteinase K to ultra-pure distilled water and 10 mM Tris-HCl) for 20 h (on a shaker at 250 rpm, 60 °C). GAG content was measured in a 96-well plate by a colorimetric DMMB (Sigma-Aldrich, Cat: 341088), using chondroitin sulfate (Sigma-Aldrich, Cat: c4384) for the standard curve. Plates were read at 530 nm wavelength on an Enspire Plate Reader. GAG content was normalized to DNA content using the Sigma-Aldrich DNA Quantification Kit (Cat: DNAQF).

### Delivery of *FOXF1* to IVD cells *in vitro* and *in vivo* through engineered EVs (Fig. 2)

#### FOXF1 transcription factor plasmid expansion

Human *FOXF1* plasmids were generated as previously described for *FOXF1* bulk transfection/reprogramming studies. Mouse *Foxf1* plasmids were obtained from Origene (Cat: MR225056, reference sequence NIH: NM_010426), expanded in *E. coli* presenting kanamycin (25 μg/mL) selectivity, isolated, and quantified.

#### NP and fibroblast cell expansion

Human NP cells from autopsy (*n* = 4) (Table 1) were expanded as described for *FOXF1* bulk transfection/reprogramming studies and pooled for downstream engineered EV generation and treatment of human diseased NP cells *in vitro*. PMEF from EMD Millipore (Burlington, MA, USA, Cat: PMEF HL) were expanded in DMEM, supplemented with 10 % FBS, 1 % antibiotic-antimycotic 100× (GIBCO, Cat: 15240062), 1 % non-essential amino acids 100× (GIBCO, Cat: 11140050), in standard conditions (5 % CO_2_, 37 °C), until they reached 80–85 % confluence for transfection.

#### Engineered vesicle generation and isolation

After expansion, human NP cells and PMEFs were resuspended at a final concentration of 1.0 × 10^6^ cells in 100 μL of electrolytic buffer per transfection group. *FOXF1* and pCMV6 (sham) plasmids solutions were prepared at 0.05 μg/μL in an electrolytic buffer. Non-viral cell transfection of the plasmids was performed by bulk electroporation using the Neon Transfection System MPK5000 following manufacturer’s instructions (1,425 V, 30 ms, 1 pulse) as described for *FOXF1* bulk transfection/reprogramming studies. After transfection, cells were cultured for 24 h in standard conditions with medium containing exosome-depleted FBS (GIBCO, Cat: A27208-01). To isolate EVs, culture medium was recollected 24 h after transfection and centrifuged at 2,000 ×*g* for 30 min at 4 °C to remove dead cells and debris, as previously reported (Duarte-Sanmiguel *et al.*, 2020; Gallego-Perez *et al.*, 2017). After centrifugation, the supernatant containing the EVs was transferred to a new tube and Total Exosome Isolation Reagent (Thermo Fisher Scientific) was added at a 1 : 2 ratio (exosome reagent : supernatant) and incubated at 4 °C overnight. Then, samples were centrifuged at 10,000 ×*g* for 1 h at 4 °C. Engineered EVs were obtained and pellets were stored at − 80 °C until characterization prior to use. EV concentration and size distribution were measured using Malvern NanoSight NS300 (ATA Scientific instruments). RT-qPCR on mRNA was performed on both the transfected cells and generated EVs to ensure successful transfection of *FOXF1* into donor cells and packaging within the engineered EVs. For imaging purposes, EVs were labeled with a red fluorescent cell linker for membrane labeling (Sigma-Aldrich, Cat: PKH26GL-1KT) following manufacturer protocol.

#### In vitro human NP cells and treatment with designer EVs

Human NP cells (*n* = 4) were seeded in 24-well plates at 50,000 cells/well in standard disc cell medium (37 °C, 5 % CO_2_). Approximately 1.5 × 10^6^ NP cells were transfected to generate 6 × 10^10^ EVs/mL to treat 50,000 NP cells in culture. After 24 h, cells were washed with sterile 1× PBS and human NP-cell-derived *FOXF1*- or pCMV6-loaded EVs (~ 1 × 10^9^ EVs resuspended in disc cell medium) were added to each well. Brightfield and fluorescent images of the cells were captured at 24 h, 48 h, and 7 d post-transfection and overlaid to visualize uptake of engineered EVs stained with the PKH26 fluorescent (red) marker. Effective delivery of *FOXF1* was assessed by quantification of *FOXF1* expression at 2 and 7 d by RT-qPCR, as described for *FOXF1* bulk transfection/reprogramming studies.

#### Pilot in vivo mouse lumbar disc puncture and designer EV injection

To assess engineered EV delivery of *Foxf1 in vivo*, a mouse lumbar IVD puncture model was used (IACUC#2016A00000074-R). 15-week old male wildtype mice (The Jackson Laboratory, Sacramento, CA, USA, Cat: C57BL/6J) were injected with 0.1 mg/kg buprenorphine SR for post-operative pain management. Mice were anesthetized using 5 % isoflurane and maintained under anesthesia (1.5–2.0 % isoflurane) on a heating pad for the entire procedure (~ 30 min). A 40 mm diameter patch of fur was removed from the mouse abdomen and the skin was sterilized following 3 cycles of 70 % isopropyl alcohol and betadine. A 1.5 cm left unilateral incision was made on the mouse abdomen and skin and organs pushed aside to reveal the lumbar spine and IVD as described in detail by Shi *et al.* (2018). Lumbar L4/L5, L5/L6, and L6/S1 discs were punctured with a 30G needle connected to a 10 μL Hamilton syringe with a plastic stopper of 1 mm needle depth. The discs of each mouse were injected with 2 μL of medical-grade saline solution containing either no EVs (injury sham), *FOXF1*-loaded EVs or pCMV6-loaded EVs (vector control) derived from PMEFs (*n* = 3 per injury control). Post injection, the injury site was sutured by nylon sutures and the mice were sacrificed 7 d post-surgery. Whole mice IVDs (including NP, AF, and endplates) were dissected and incubated with Hoechst and calcein-AM. Fluorescent images of the IVDs were captured to qualitatively assess cell viability. To quantify the success of delivery, RT-qPCR performed for *FOXF1* and *Brachyury* was performed on the entire disc as described previously with relevant whole disc controls.

#### Statistical analysis

To compare differences between bulk transfection of *FOXF1 vs*. pCMV6 vector controls and FOXF1-loaded EVs *vs*. pCMV6-loaded EVs, non-parametric, two-tailed, unpaired Mann-Whitney tests were performed for α = 0.05, with a Bonferroni correction for multiple comparisons. For *in vivo* mouse studies comparing gene expression in injury, pCMV6, and *Foxf1* treatment groups, a one-way ANOVA was used followed by Tukey *post-hoc* test with α = 0.05.

## Results

### *In vitro* reprogramming of human NP cells by bulk electroporation of *FOXF1*

#### Cell viability

**Fig 3.**
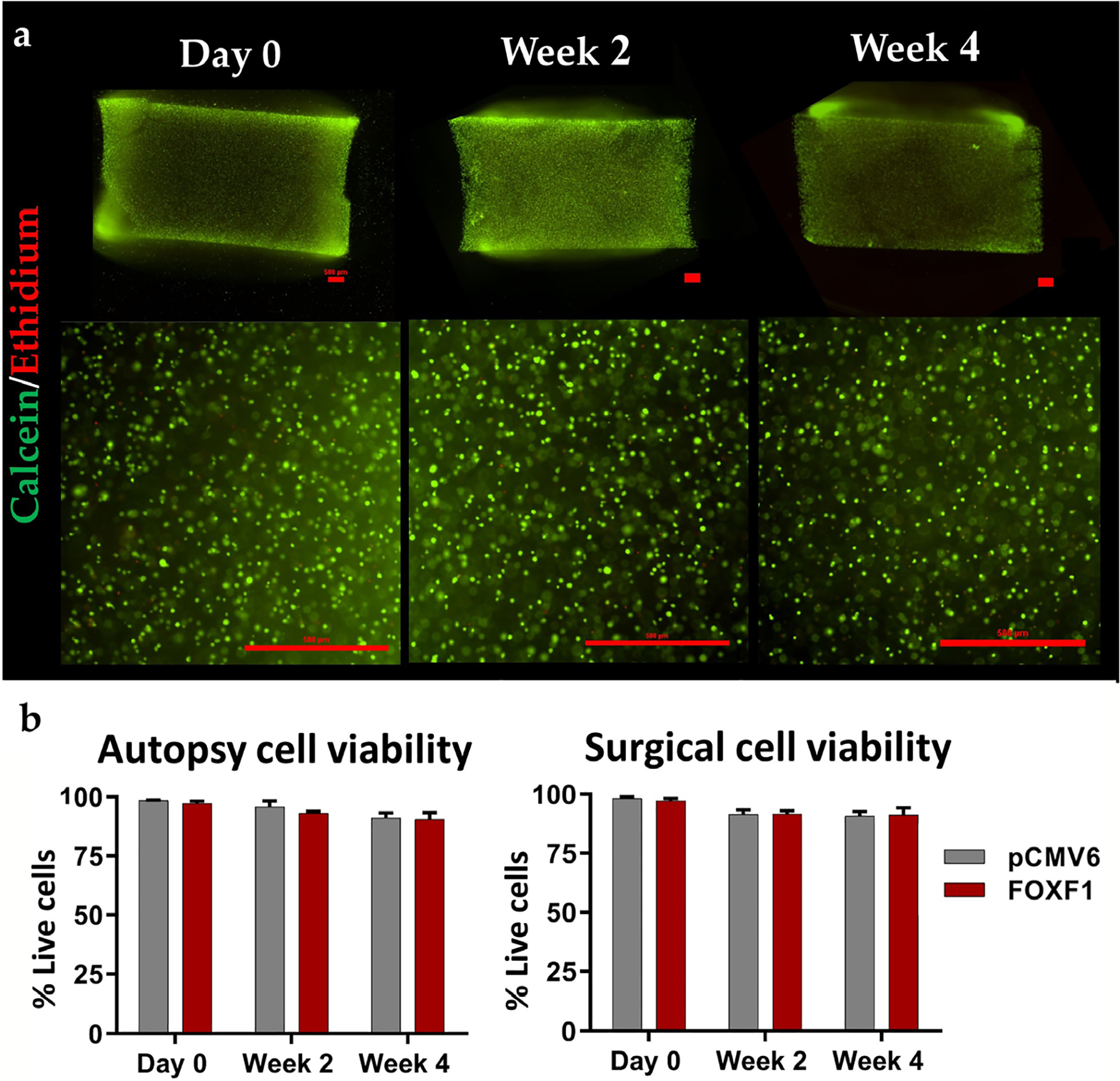
Cell viability of 3D gels. (**a**) Representative 4× (top) and 10× images (bottom) of calcein-AM (live) and ethidium-homodimer (dead) stained sagittal cryosections of cell-embedded 3D agarose gels at day 0, week 2 and 4 weeks in culture (scale bar: 500 μm). Brightness of images adjusted equally for each image for better contrast. (**b**) Quantified cell viability based on number of live cells over total cells of respective FOXF1 and pCMV6 transfected groups for autopsy and surgical cells. No statistical differences were observed between groups.

To access the viability of human NP cells in the 3D construct, a live/dead assay was used that stained live cells green (calcein-AM) and dead cells red (ethidium-homodimer). No significant differences in cell viability were observed for pCMV6- and *FOXF1*-transfected groups across the 4-week culture in autopsy and surgical NP cells, and all constructs maintained more than 90 % viability (Fig. 3a,b).

#### Gene expression: phenotypic markers, inflammatory cytokines, NGF, matrix enzymes

RT-qPCR was used to quantify the relative fold change in gene expression of healthy NP markers, inflammatory cytokines, *NGF*, and matrix enzymes in human NP cells transfected with *FOXF1*, normalized to pCMV6-transfected control NP cells and the housekeeping gene *18S*. All comparisonns discussed below are between *FOXF1*-treated and pCMV6 control.

**Fig 4.**
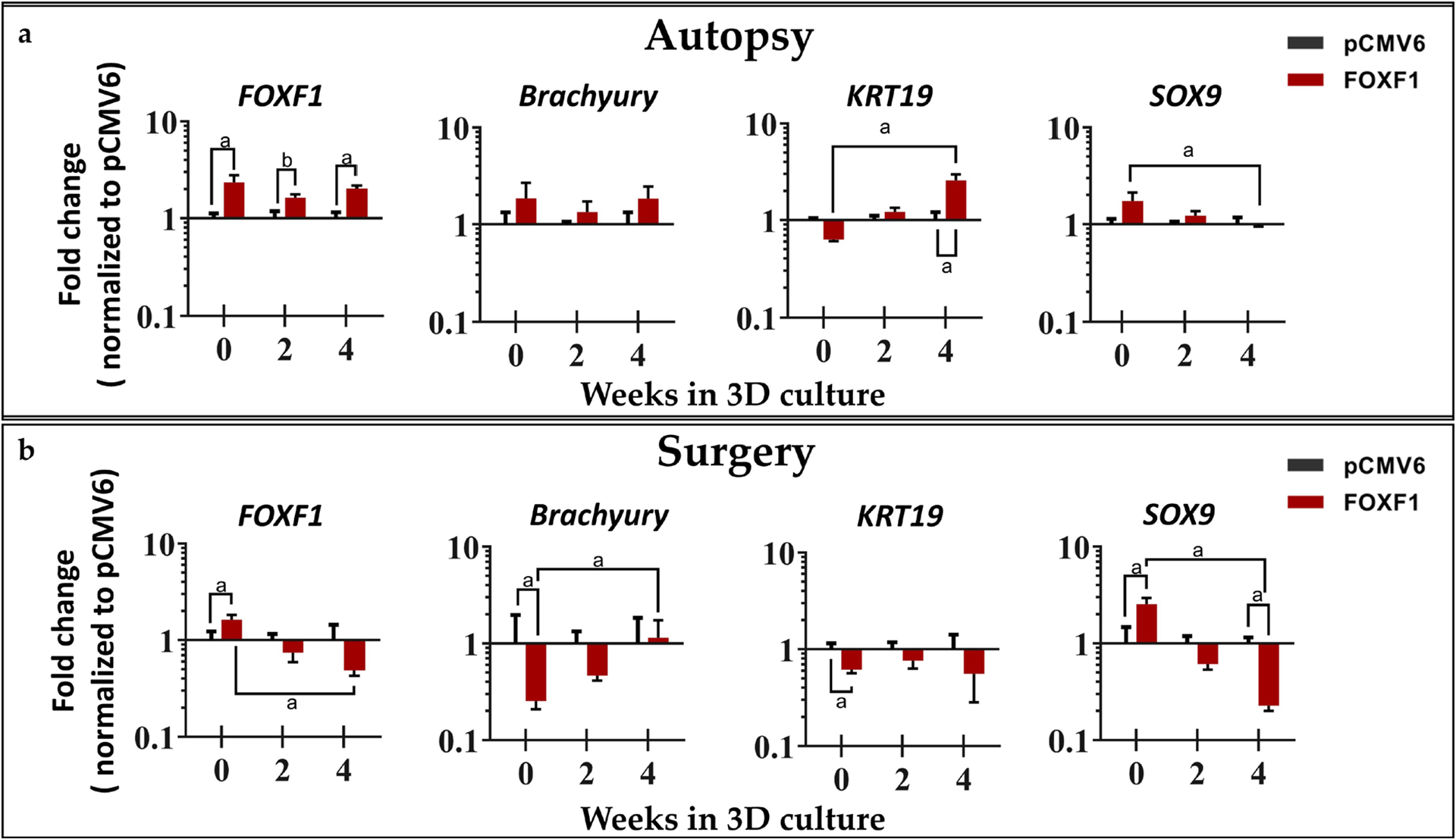
Phenotypic marker gene expression. *FOXF1*, *Brachyury*, *KRT19*, and *SOX9* for (**a**) autopsy (*n* = 5) and (**b**) surgical human NP cells (*n* = 5) at 0, 2, and 4 weeks in 3D agarose culture normalized to pCMV6 controls and house-keeping gene *18S* (^a^ *p* < 0.05, ^b^ *p* = 0.0556 for *FOXF1*-treated NP cells when compared to pCMV6 controls).

The healthy NP marker *FOXF1* was upregulated at all time points at day 0 = 2.35-fold (*p* = 0.0159), week 2 = 1.64-fold (*p* = 0.0556), and week 4 = 2.03-fold (*p* = 0.0079) in autopsy NP cells, while only significantly upregulated at day 0 (1.63-fold, *p* = 0.0493) in surgical NP cells. Although no significant differences were found in *Brachyury* for autopsy NP cells, significant downregulation was observed at day 0 (− 3.93-fold, *p* = 0.0079) for surgical NP cells. *KRT19* was upregulated at week 4 (2.59-fold, *p* = 0.0079) in autopsy NP cells, with downregulation in surgical NP cells at day 0 (− 1.61-fold, *p* = 0.0159). Lastly, SRY-box transcription factor 9 (*SOX9*), a chondrocyte marker, showed no significant differences in autopsy NP cells but was upregulated at day 0 (2.56-fold, *p* = 0.0159) in surgical NP cells and downregulated at week 4 (− 4.39, *p* = 0.0079) (Fig. 4).

**Fig 5.**
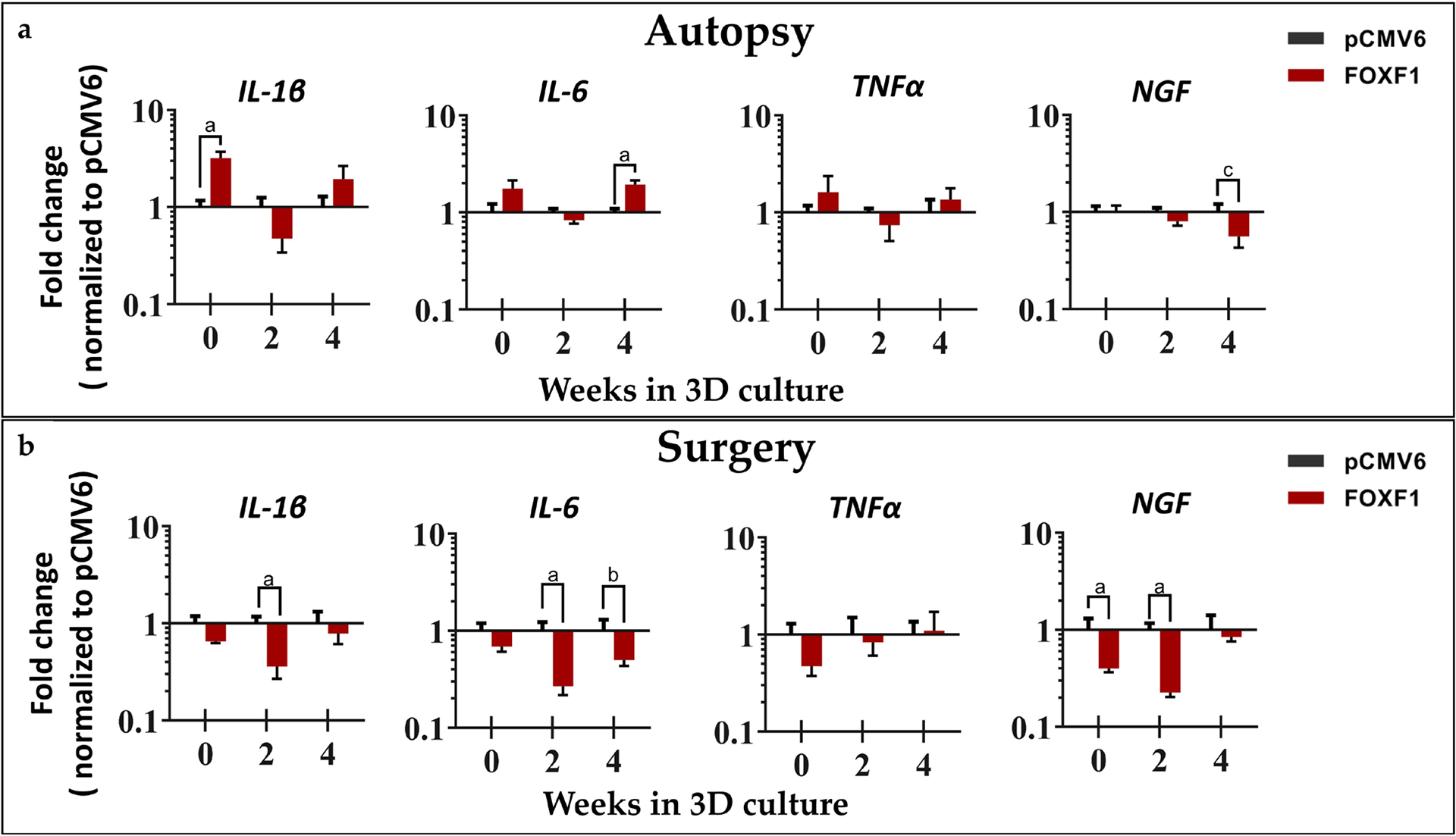
Inflammatory cytokine and NGF gene expression. *IL-1β*, *IL-6*, *TNF-α* and *NGF* for (**a**) autopsy (*n* = 5) and (**b**) surgical human NP cells (*n* = 5) at 0, 2, and 4 weeks in 3D agarose culture normalized to pCMV6 controls and house-keeping gene *18S* (^a^ *p* < 0.05, ^b^ *p* = 0.0873, and ^c^ *p* = 0.079 for *FOXF1*-treated NP cells when compared to pCMV6 controls).

Inflammatory cytokine *IL-1β* was upregulated in autopsy NP cells at day 0 (3.20-fold, *p* = 0.0079), while significantly downregulated in surgical NP cells at week 2 (− 2.78-fold, *p* = 0.0317). Similarly, *IL-6* was upregulated at week 4 in autopsy NP cells (1.94-fold, *p* = 0.0079) but significantly downregulated at week 2 (− 3.73-fold, *p* = 0.0079) in surgical NP cells. No significant differences were observed for *TNF-α* in either autopsy or surgical NP cells. *NGF* was significantly downregulated at day 0 (− 2.49-fold, *p* = 0.0159) and week 2 (− 4.42-fold, *p* = 0.0079) in surgical NP cells (Fig. 5).

**Fig 6.**
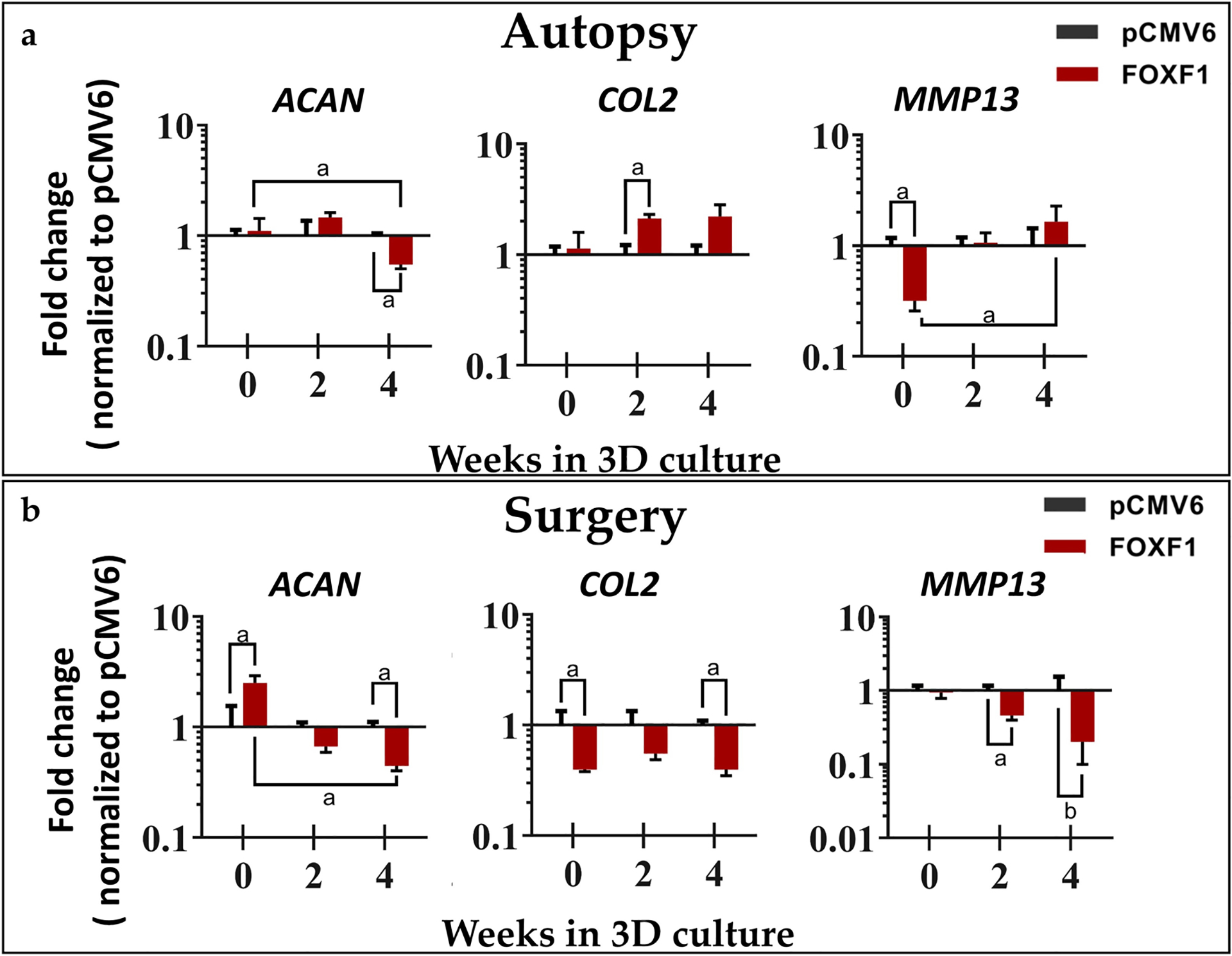
Matrix gene expression for anabolic genes *ACAN* and *COL2* and catabolic *MMP13*. (**a**) Autopsy (*n* = 5) and (**b**) surgical (*n* = 5) human NP cells at 0, 2, and 4 weeks in 3D agarose culture respectfully normalized to pCMV6 controls and house-keeping gene *18S* (^a^ *p* < 0.05, ^b^ *p* = 0.0556 for *FOXF1*-treated NP cells as compared to pCMV6 controls).

Matrix gene *ACAN* was downregulated at 4 weeks (− 1.81-fold, *p* = 0.238) for autopsy NP cells but was significantly upregulated at day 0 (2.50-fold, *p* = 0.0476) and downregulated at 4 weeks (2.23-fold, *p* = 0.0079) in surgical NP cells. *COL2* was significantly upregulated at week 2 (2.12-fold, *p* = 0.0159) in autopsy NP cells, but significantly downregulated at both day 0 (− 2.52-fold, *p* = 0.0079) and week 4 (− 2.51-fold, *p* = 0.0079) in surgical samples. *MMP13* expression was significantly decreased at day 0 (− 3.16-fold, *p* = 0.0079) in autopsy NP cells. In surgical cells, *MMP13* showed significant downregulation at both 2 (− 2.17-fold, *p* = 0.0317) and 4 weeks (− 4.93-fold, *p* = 0.0556) (Fig. 6).

#### GAG content

**Fig 7.**
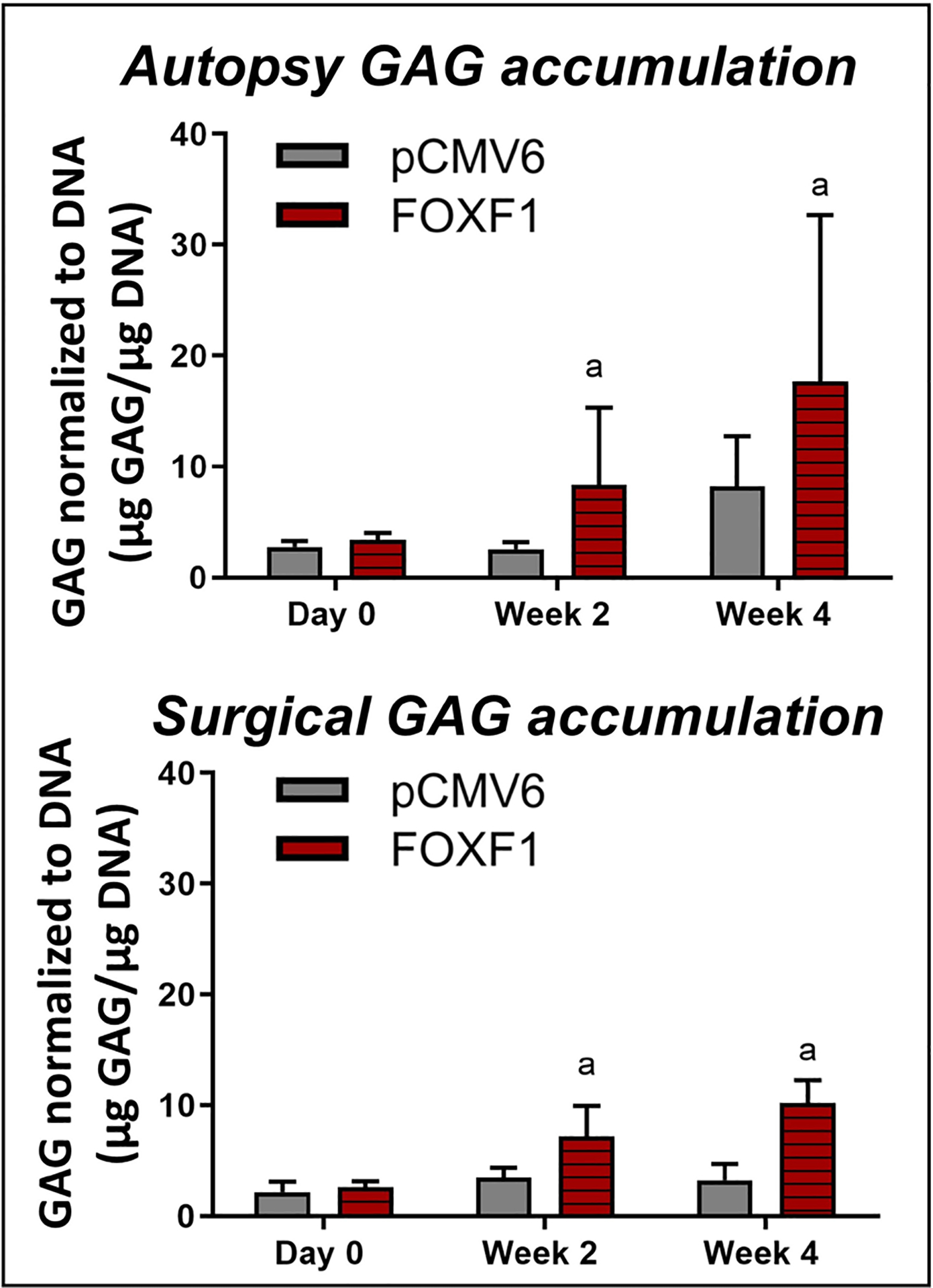
GAG accumulation. Normalized to DNA content of autopsy (top, *n* = 5) and surgical (bottom, *n* = 5) human NP cells in 3D culture at 0, 2 and 4 weeks (^a^ *p* < 0.05 for *FOXF1*-transfected and pCMV6 control).

No significant differences in GAG content were observed at day 0; however, significantly increased GAG accumulation was observed at week 2 (8.32 ± 7.00 μg GAG/μg DNA, *p* = 0.0413) and week 4 (17.68 ± 15.05 μg GAG/μg DNA, *p* = 0.0079) in *FOXF1*-transfected autopsy NP cells when compared to pCMV6 controls. Similarly, no significant differences in GAG content were observed at day 0 for surgical NP cells, but significantly increased GAG accumulation was observed at week 2 (7.18 ± 2.68 μg GAG/μg DNA, *p* = 0.0357) and week 4 (10.18 ± 2.06 μg GAG/μgDNA, *p* = 0.0079) in *FOXF1*-transfected surgical NP groups when compared to pCMV6 controls. No significant difference in raw DNA content was observed (Fig. 7).

### Delivery of FOXF1 to IVD cells *in vitro* and *in vivo* through engineered EVs

#### Validation of engineered EV generation

**Fig 8.**
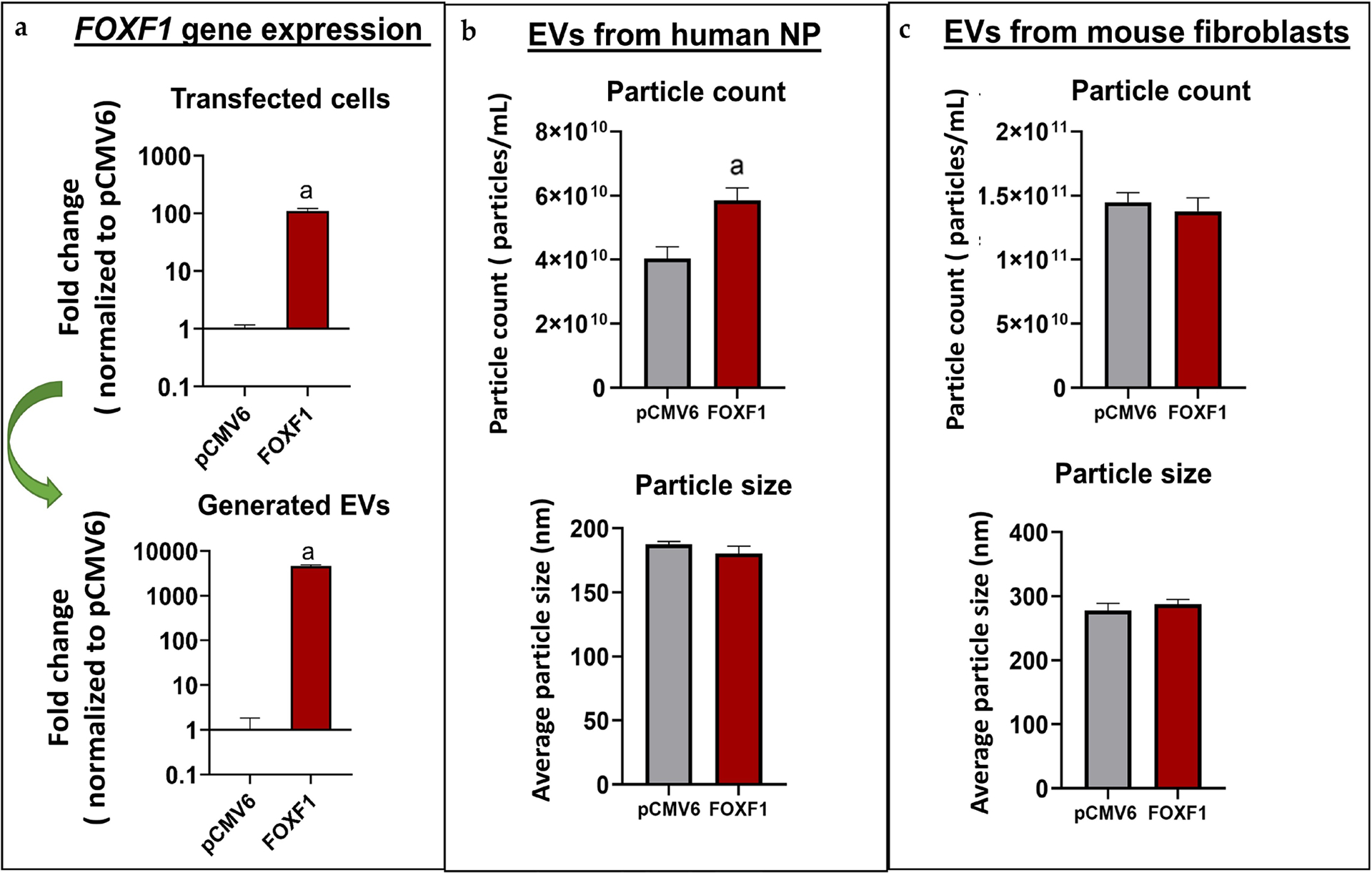
EV characterization. (**a**) *FOXF1* expression in transfected human NP cells (top) and generated EVs (bottom) in FOXF1 groups normalized to pCMV6 (*n* = 4). (**b**) Average particle count and particle size quantified by NanoSight NS300 for EVs generated from human NP cells. (**c**) Average particle count and particle size quantified by NanoSight NS300 for EVs generated from mouse embryonic fibroblasts (^a^ *p* < 0.05).

NP cells transfected with *FOXF1* demonstrated a significant increase in FOXF1 expression (109.89-fold, *p <* 0.01) as compared to NP cells transfected with pCMV6 (Fig. 8a). Similarly, engineered EVs loaded with *FOXF1* demonstrated significant increases in *FOXF1* expression (4585.63-fold, *p <* 0.01) as compared to EVs loaded with pCMV6, highlighting successful transfection of *FOXF1* and loading of engineered EVs with *FOXF1*. *FO*X*F1*- and pCMV6-loaded EVs generated from human NP cells demonstrated significant differences in particle count (5.85 × 10^10^ particles/mL and 4.03 × 10^10^ particles/mL for *FOXF*1 and pCMV6, respectively, *p* = 0.028), with no significant difference in particle size (average = 184 nm) (Fig. 8b). Engineered EVs generated from PMEFs and loaded with *FOXF1* or pCMV6 for *in vivo* studies showed no significant difference in either particle count (1.41 × 10^11^ particles/mL) or size distribution, with an average particle size of 282 nm (Fig. 8c).

#### Designer EV uptake by NP cells in monolayer

**Fig 9.**
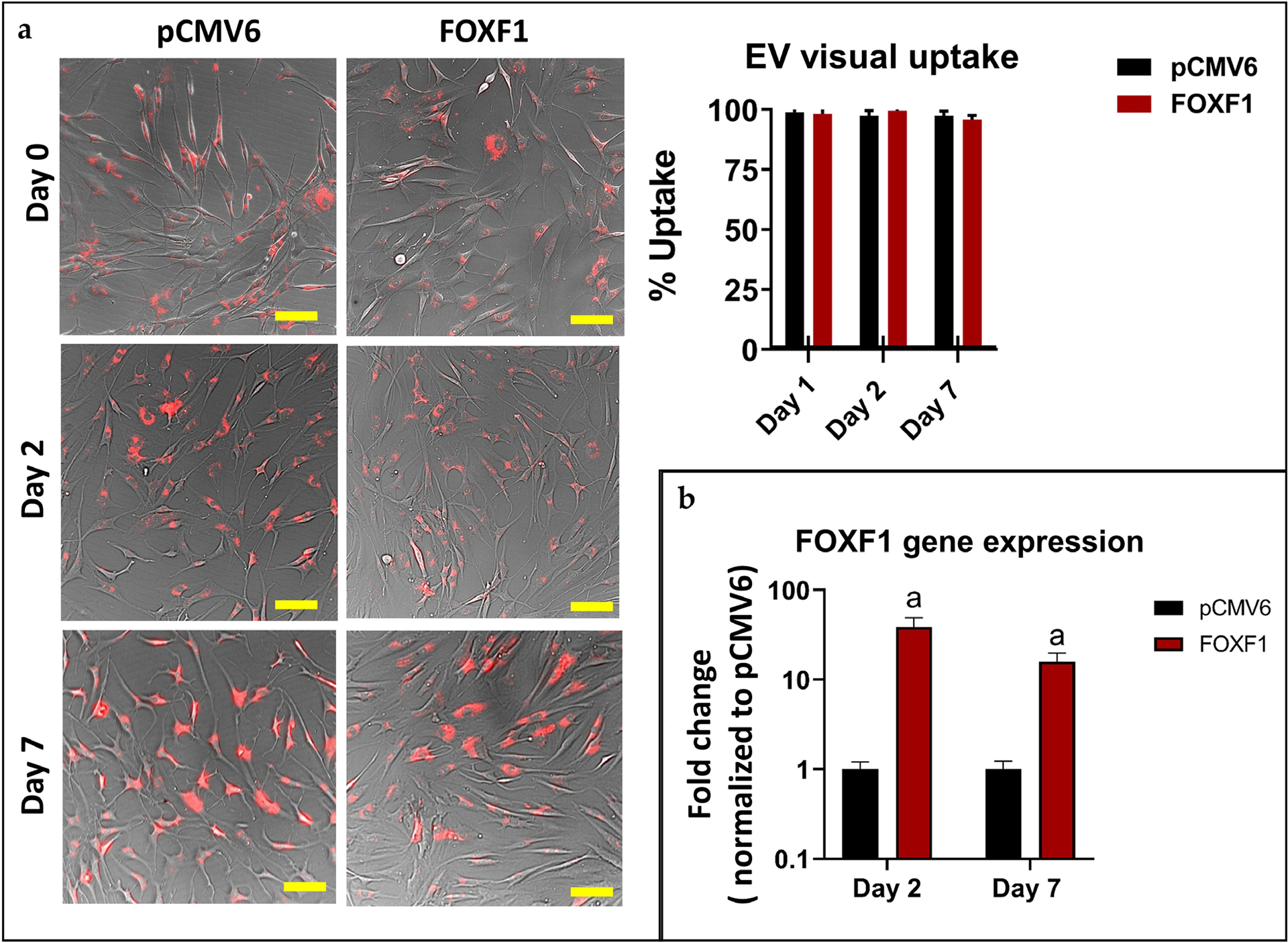
EV uptake by cells. (**a**) Brightfield images of NP cells co-cultured in monolayer with PKH26-stained pCMV6 and *FOXF1* EVs (red) respectively at 0, 2, and 7 d along with quantified visual uptake by percentage of cells with EVs over total cells. (**b**) *FOXF1* expression of *FOXF1*-EV-treated human NP cells in monolayer normalized to pCMV6-EV-treated cells (^a^ *p* < 0.05).

Brightfield images of NP cells in monolayer were overlaid with fluorescent images of PHK26-stained EVs and the percentage of EV uptake was quantified by the number of cells containing EVs (stained red) over the total number of cells imaged. Combined images demonstrated more than 95 % uptake of stained EVs for both *FOXF1* and pCMV6 groups at day 1, 2, and 7, with no significant differences between time points (Fig. 9a). Gene expression for *FOXF1* was assessed, with significant upregulation of *FOXF1* in cells treated with *FOXF1*-loaded EVs *vs*. pCMV6 EVs (control)-treated cells at both day 2 (38.53-fold, *p <* 0.0286) and 7 (15.82-fold, *p* < 0.0286) (Fig. 9b).

#### Cell viability in IVDs injected with EVs

**Fig 10.**
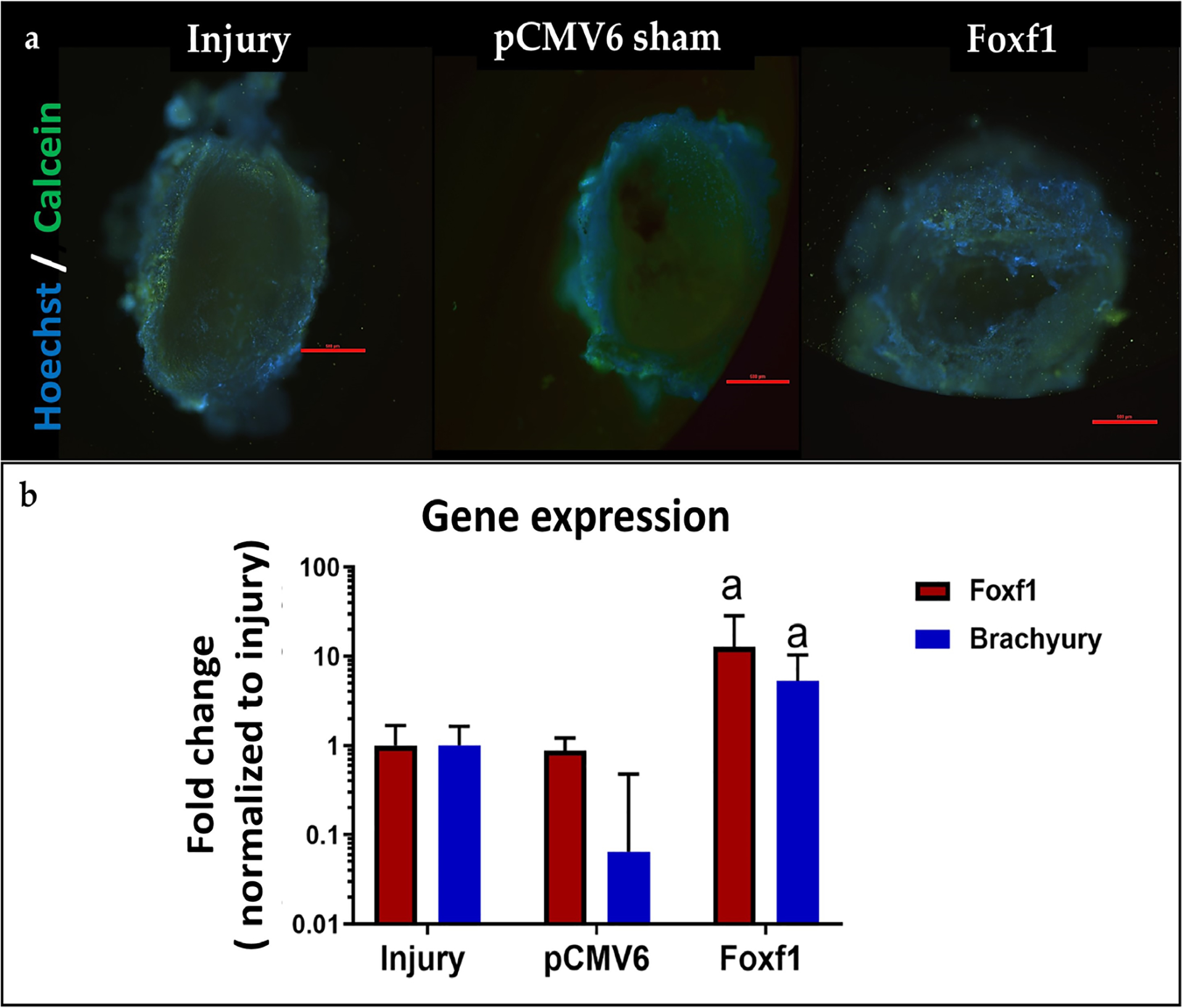
EV uptake by cells. (**a**) Representative fluorescent images of injury, pCMV6, and *Foxf1*-EV-treated mouse IVDs dissected post euthanasia and stained with Hoechst (DNA/nucleus) and calcein-AM (live/cytosol) (scale bar: 500 nm). (**b**) *Foxf1* and *Brachyury* expression of mice IVD 7 d post-injection for Foxf1-and pCMV6-treated mice normalized to injury controls and house-keeping gene *18S* (^a^ *p* < 0.05).

Mice IVDs were harvested after euthanasia at 7 d post-treatment with engineered EVs or saline and stained with Hoechst (blue = DNA) and calcein-AM (green = live cell) to qualitatively assess cell viability. No qualitative differences were observed between injury, pCMV6, and *Foxf1*-loaded EVs groups, across all isolated discs (Fig. 10a). Due to the high cell density and the 3D structure of the disc, images could not be quantified.

#### Gene expression in mice discs suggestive of successful EV uptake

RT-qPCR was used to assess gene expression of *Foxf1* and *Brachyury* in discs injected with *Foxf1-* or pCMV6-loaded engineered EVs. Data were normalized to injury controls and the housekeeping gene *18S*. *Foxf1* was significantly upregulated in disc cells treated with *Foxf1*-loaded EVs when compared to the injury control group (*p* = 0.048). *Brachyury* was also significantly upregulated in disc cells treated with *Foxf1*-loaded EVs when compared to the injury control group (*p* = 0.049). No significant differences were identified in either gene between disc cells treated with pCMV6- loaded EVs and the injury control group (*p* > 0.05) (Fig. 10b). Phenotypic markers, inflammatory cytokines, and matrix markers were also explored for the mice IVDs but no significant differences were found.

## Discussion

The study findings demonstrated that FOXF1 can reprogram human NP cells from autopsy and surgery to a healthier anti-catabolic and anti-inflammatory state. Furthermore, the study also demonstrated that engineered EVs can be used as a non-viral delivery system to deliver transcription factors, such as *FOXF1*, to human NP cells *in vitro* and to mouse IVD cells *in vivo*, with limited cytotoxicity and effective up-regulation of genes of interest. The study suggested that harnessing potential developmental transcription factors, such as FOXF1, to reprogram degenerate NP cells back to a healthy state could be a viable therapeutic strategy for treating IVD degeneration and associated discogenic pain. Significant increases in GAG accumulation were observed together with decreased expression of inflammatory cytokines and matrix degradation enzyme, such as MMP13, all hallmarks of a healthy IVD. Furthermore, the study was the first to demonstrate a successful non-viral gene delivery to IVD cells both *in vitro* and *in vivo* using engineered EVs, highlighting the potential of this intervention to treat IVD degeneration and discogenic back pain.

Previously, Tang *et al.* (2019) and Vujovic *et al.* (2006) have demonstrated the potential of the transcription factor Brachyury to reprogram and regenerate the degenerate IVD due to its elevated levels in the immature healthy NP and its role in notochord development. In the present study, a novel role for *FOXF1* was investigated due to its high expression in the healthy NP, its regard as an NP specific marker, and its roles in cell proliferation, differentiation, and growth (Richardson *et al.*, 2017b; Risbud *et al.*, 2015). First, the regenerative potential of *FOXF1* was investigated in an *in vitro* study using bulk electroporation. Assessment of cell viability demonstrated no significant differences between all groups, suggesting that bulk electroporation of *FOXF1* or pCMV6 (control) plasmids had no detrimental effects on human NP cells, similar to bulk transfection with *Brachyury*. Successful transfection of *FOXF1* in human NP cells was determined by assessing *FOXF1* expression in the transfected human NP cells from autopsy and surgery when compared to pCMV6 controls. FOXF1 was significantly up-regulated at all time-points for autopsy NP cells; however, a more temporal response was observed for surgical NP cells, indicating that *FOXF1* was successfully transfected to the NP cells but tissue source and degenerative conditions may affect transfection and reprogramming efficiency. In comparison, bulk electroporation of *Brachyury* at the same plasmid concentration into autopsy and surgical NP cells showed a much larger fold change (~ 100-fold) *vs*. empty vector controls (Tang *et al.*, 2019). A similar study using bulk electroporation of human MSCs with GDF5 demonstrated upregulation of GDF5 up to 1,000-fold in 3D alginate culture (Bucher *et al.*, 2013). An additional study transfected primary human NP cells with GDF6 and found increased expression of GDF6 over 14 d (May *et al.*, 2017).

With the validation of successful transfection of FOXF1, healthy phenotypic, inflammatory, and neurotrophic markers were also assessed. Brachyury and KRT19 are considered to be healthy NP markers and present in the immature NP; also, the chondrogenic marker SOX9 has been shown to increase matrix production in the human IVD (Lee *et al.*, 2007; Minogue *et al.*, 2010; Paul *et al.*, 2003; Risbud *et al.*, 2015). Therefore, they are excellent candidate markers for assessing successful reprogramming of degenerate NP cells to a healthy NP-like phenotype. Unfortunately, significant upregulation of Brachyury was not observed in either autopsy or surgical cells, with downregulation of *Brachyury* expression at day 0 in surgical NP cells. Brachyury is a regulator of mesodermal FOXF1 (Zhou *et al.*, 2018) and this has also been demonstrated in human NP cells electroporated with Brachyury (Tang *et al.*, 2019). However, no effect of *FOXF1* on *Brachyury* expression were observed. *KRT19* was upregulated at week 4 in autopsy NP cells, with downregulation in surgical NP cells, while *SOX9* was upregulated at day 0 for autopsy and surgical NP cells but expression was decreased at both weeks 2 and 4 when compared to controls. This suggested that the effects of *FOXF1* transfection from bulk electroporation are transient and that the degree of degeneration between the less degenerate autopsy samples and painful degenerate surgical samples likely influences these effects and transfection efficiency.

IVD degeneration is also characterized by elevated levels of inflammatory cytokines (*IL-1β*, *IL-6*, *TNF-α*) and *NGF* expression (Freemont *et al.*, 1997; Le Maitre *et al.*, 2007b; Purmessur *et al.*, 2008; Shamji *et al.*, 2010). Therefore, the downregulation of these markers may indicate decreased inflammation and nerve ingrowth. *IL-1β* and *IL-6* were downregulated at week 2 and weeks 2 and 4, respectively, in surgical NP cells, yet interestingly upregulated in autopsy NP cells when compared to pCMV6 sham controls. This demonstrated that *FOXF1* may downregulate inflammatory cytokines in degenerate NP cells over time, however to a lesser extent in NP cells from autopsy. There were no significant differences in *TNF-α* expression, suggesting that *FOXF1* has minimal effect on *TNF-α* when compared to pCMV6 controls. Neurotrophic pain marker *NGF* was downregulated in autopsy NP cells at week 4 and surgical NP cells at day 0 and week 2, suggesting a reduced ability of NP cells to promote neo-innervation, as nerve ingrowth and NGF are upregulated in painful IVDs (Freemont *et al.*, 2002).

High levels of aggrecan and collagen II are key hallmarks of the healthy NP, however in degeneration, increased ECM degradation and decreased matrix synthesis are observed, leading to a loss of IVD structure and mechanical function (Ghosh, 1988). Therefore, increasing matrix synthesis while decreasing catabolic gene expression is critical for NP regeneration. In the present study, the matrix genes *ACAN* and *COL2* were assessed along with the matrix enzyme *MMP13*. *MMP13* was downregulated at day 0 in autopsy NP cells while downregulated at weeks 2 and 4 in surgical NP cells in *FOXF1*-transfected groups normalized to pCMV6 control, suggesting decreased matrix catabolism in human NP cells. Surprisingly, *ACAN* was downregulated in autopsy and surgical cells at week 4 and upregulated at day 0 in surgical NP cells only. *COL2* exhibited upregulation at week 2 in autopsy NP cells but was consistently downregulated in surgical NP cells. In comparison, on the protein level, GAG accumulation increased in *FOXF1*-treated groups as compared to pCMV6 at both weeks 2 and 4. As a major hallmark of IVD degeneration is the loss of GAG, increased GAG accumulation is a major indicator of reprogramming success towards a healthy NP phenotype. The inconsistency in gene-level *vs*. GAG protein measurements could be explained by the temporal nature of RT-qPCR as a snapshot in time, as seen in a previous study (Tang *et al.*, 2019). Notably, May *et al.* (2017) also found no significant difference in *ACAN* and *COL2* expression after electroporation of *GDF6*.

Bulk electroporation of *FOXF1* into autopsy and surgical NP cells can reprogram human NP cells based on gene-level assessments and notable protein level GAG expression. However, bulk electroporation has limited clinical relevance due to challenges associated with apoptosis, necrosis, and cellular dysfunction of cells as well as translation *in vivo* (Batista Napotnik *et al.*, 2016). For this reason, the present study did not utilize a control (non-bulk-electroporated) group since the aim was to focus only on the effects of FOXF1 on the cells, not bulk electroporation, as it lacks clinical relevance. Thus, a more clinically relevant non-viral gene delivery method was desired and, therefore, engineered EVs were investigated. EVs are nanoscale particles composed of a lipid bilayer membrane that is secreted by all cell types and can carry lipids, proteins, and genetic material, such as RNA and DNA (O’Brien *et al.*, 2020). One of the advantages of using EVs is that they can be derived from a variety of sources in a considerable amount, including many different tissues and biofluids (saliva, plasma, breast milk, urine, cerebrospinal fluid). They have the potential to be produced in large quantities, include cell-specific surface markers of the derivative cell, and are biocompatible and stable for long-term storage (Murphy *et al.*, 2019; O’Brien *et al.*, 2020).

Engineered EVs offer many advantages when compared with other delivery methods – such as synthetic nanocarriers, viral vectors, and non-viral physical and chemical transfection methods – including the ability to penetrate biological barriers, to be functionalized for targeted delivery, and pack diverse molecular cargo, improved stability in biofluids and circulation, as well as decreased probability of inducing adverse effects (Gantenbein *et al.*, 2020). Moreover, the therapeutic potential of EVs has been a growing topic of interest and the investigators have previously explored their role in delivery and reprogramming of nerve and blood vessels (Gallego-Perez *et al.*, 2017). In IVD degeneration, EVs derived from MSCs have been investigated mainly in terms of their innate therapeutic potential and delivery of microRNAs such as miR-21 (Cheng *et al.*, 2018; Piazza *et al.*, 2020). However, the potential of non-viral delivery of exogenous transcription factors such as *FOXF1* using designer EVs in diseased NP cells remains unexplored.

Bulk electroporation was used to generate *FOXF1*- and sham-loaded designer EVs from human NP cells and primary mouse embryonic fibroblasts. High *FOXF1* expression in transfected cells was confirmed followed by validation of effective *FOXF1* packing in the engineered EVs. Further characterization of EV size distribution and concentration demonstrated that billions of EV particles were generated from human NP-derived and mouse fibroblast cells and particle size was consistent with the literature. This analysis confirmed that enough EVs were generated for effective treatment of human cells and mice IVDs *in vivo*. The difference in particle count between pCMV6 and *FOXF1* EVs generated from human NP cells could be a potential effect of FOXF1 upregulation in the secretory capacity of transfected cells. This increase in EV production by human NP cells transfected with *FOXF1* when compared to the sham may be due to an enhanced secretory capacity of these types of cells when transfected with the transcription factor of interest.

Both *FOXF1*- and pCMV6-loaded designer EVs tagged with PKH26GL membrane marker demonstrated significantly high levels of uptake in a short-term 7 d study. As the designer EVs for the human monolayer study were derived from human NP cells, the generated EVs may exhibit preferential uptake by NP cells due to NP membrane specific markers targeted to NP cells. This highlights the potential advantage of non-viral gene delivery through designer EVs but needs to be validated in future studies. Analysis of gene expression for the *FOXF1*-loaded EV group showed significant overexpression of *FOXF1* at day 2 and 7 when compared to pCMV6 EVs, indicating successful and highly effective delivery of *FOXF1* into NP cells. Notably, EVs have also shown attraction and binding to fibronectin and collagen type I, alluding to potential retention in IVD tissue (Narayanan *et al.*, 2016). As far as it can be ascertained, there are no other reports describing the non-viral delivery of molecular cargo to the IVD through designer EVs. Other studies have assessed the content and function of non-engineered IVD-derived, MSC-derived, or notochordal-cell-derived EVs for treatment of IVD degeneration and these particular EVs have also been assessed as disease biomarkers (*e.g.* microRNAs) (Bach *et al.*, 2017; Chen *et al.*, 2018; Park *et al.*, 2019). As cell survival and functionality may limit the therapeutic potential of MSC treatments, recent literature has focused on the use of MSC-derived EVs for the reversal of disc degeneration (Huang *et al.*, 2013; Park *et al.*, 2019). These studies highlight the potential of EVs therapies as a novel therapeutic strategy to target low-back pain and IVD degeneration.

To assess the delivery of *Foxf1* to IVD tissue *in vivo* using mouse embryonic fibroblast-derived designer EVs, a pilot study using a mouse lumbar disc injury model was performed and viability, including transcription factor expression, determined at 7 d. Hoechst and calcein-AM staining of mice lumbar IVDs found no qualitative differences in IVD cell viability, suggesting that the engineered EVs have minimal cytotoxic effects on the IVD. Upregulation of Foxf1 was observed in mice discs treated with *Foxf1*-loaded EVs as compared to those treated with pCMV6-loaded EVs and injury controls. This suggested that *Foxf1* was successfully delivered to the disc and expressed for at least 7 d, but further work is required to quantify the efficiency of EV uptake and confirm Foxf1 protein expression within the disc. Interestingly, *Brachyury* was also upregulated in *Foxf1*-treated groups indicating that *Foxf1* may have a regulatory role on *Brachyury* unlike the observations for *in vitro* bulk transfection of *FOXF1*. Other phenotypic markers such as *KRT19*, along with inflammatory cytokines and matrix gene expression showed no significant differences between *Foxf1*-treated groups and controls, suggesting the need for more replicates as only *N* = 3 were used for the pilot study. These differences in effects between the *in vitro* and *in vivo* models may be attributed to several factors, for example electroporation *vs*. EV delivery, human *vs*. mouse, or the nature of an isolated *in vitro* model *vs*. a more complex *in vivo* microenvironment.

Delivery of *Foxf1* through engineered EVs showed successful transport of Foxf1 and gene expression *in vivo* over 7 d. However, longer-term *in vivo* studies are required to validate the safety, efficacy, and clinical potential of this strategy for the degenerate IVD and DBP. For example, exploration of several relevant inflammatory cytokines, as discussed in the *in vitro* studies, along with structure/function and pain behaviors. Furthermore, cells from female IVD specimens were used for the monolayer studies while male mice were used for *in vivo* studies and sex differences have been reported to be important when assessing therapeutic potential candidate targets (Mosley *et al.*, 2019; Richards *et al.*, 2019). Future studies will focus on the long-term therapeutic effects of transcription factor encased EVs with sex differences included as a factor in the analysis. In addition, further pre-clinical validation using engineered EVs with transcription factors as a therapeutic strategy for IVD degeneration and low-back pain is warranted in clinically relevant large animal models such as the chondrodystrophic canine.

## Conclusions

This is the first study to demonstrate the therapeutic potential of the developmental transcription factor *FOXF1* to reprogram human NP cells towards a healthy NP-like phenotype as demonstrated by enhanced expression of NP phenotypic markers, decreased catabolism/inflammation, and GAG accumulation using non-viral methods. Furthermore, the potential of engineered EVs to effectively deliver a plasmid encoding *FOXF1* to NP cells was shown both in monolayer *in vitro*, and in mice lumbar IVDs *in vivo*. This approach has high clinical relevance as it is minimally invasive, non-addictive, non-viral and has the potential to reprogram native degenerate IVD cells *in situ* for the treatment of discogenic back pain.

## Figures and Tables

**Fig 1.**
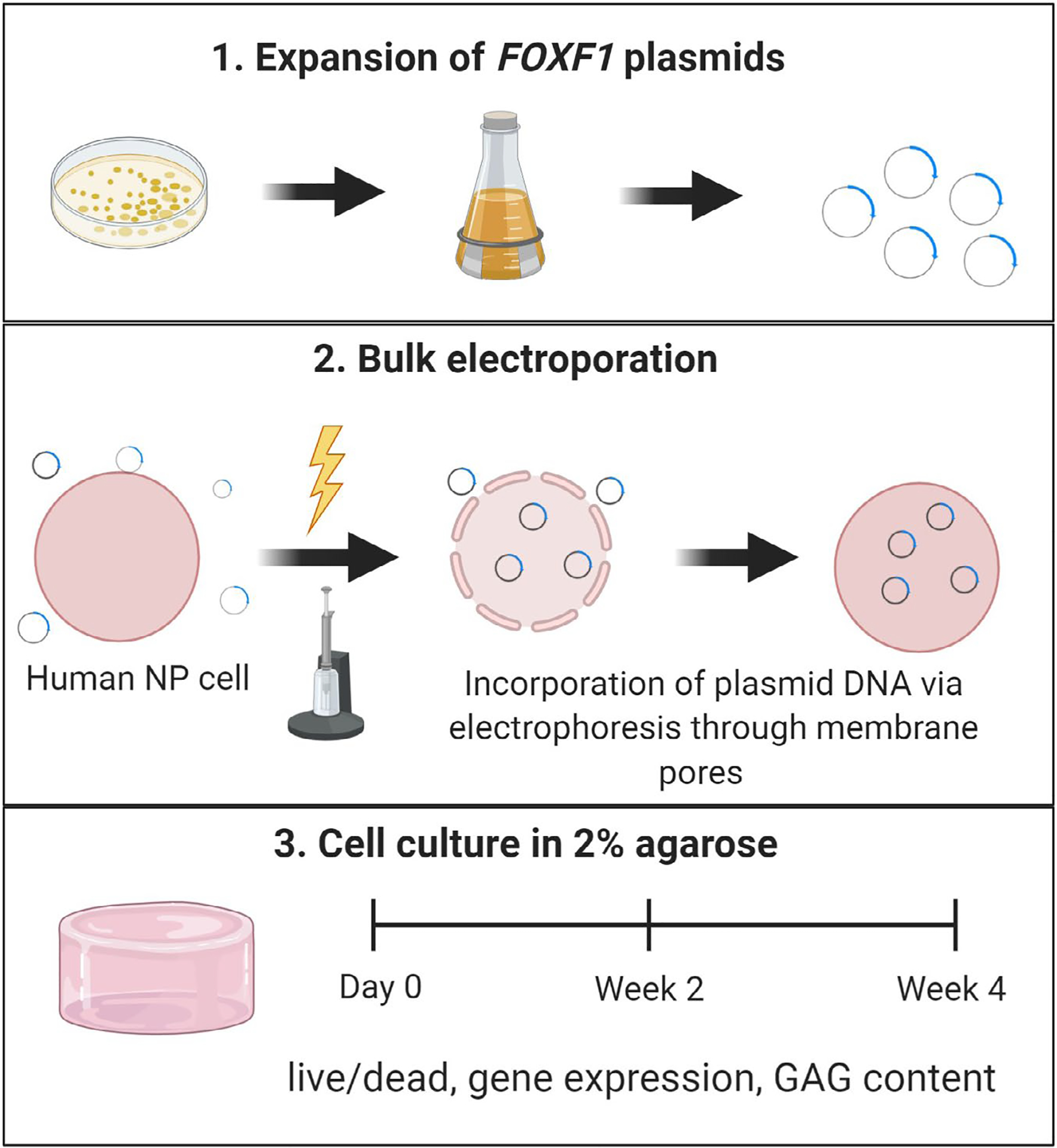
Schematic representation of *in vitro* reprogramming of human NP cells by bulk electroporation of *FOXF1*. (1) First *FOXF1* plasmids were expanded in solid agarose culture followed by large culture and isolation of expanded plasmids. (2) Plasmids were suspended in solution with human NP cells and bulk-electroporated to induce transient membrane openings. Then, plasmids infiltrated the porous cell and were enclosed inside the cell. (3) Next, electroporated cells were seeded in a 3D 2 % agarose culture for 4 weeks with dependent variables assessed at day 0, week 2, and week 4. Illustration created with licensed BioRender.com software.

**Fig 2.**
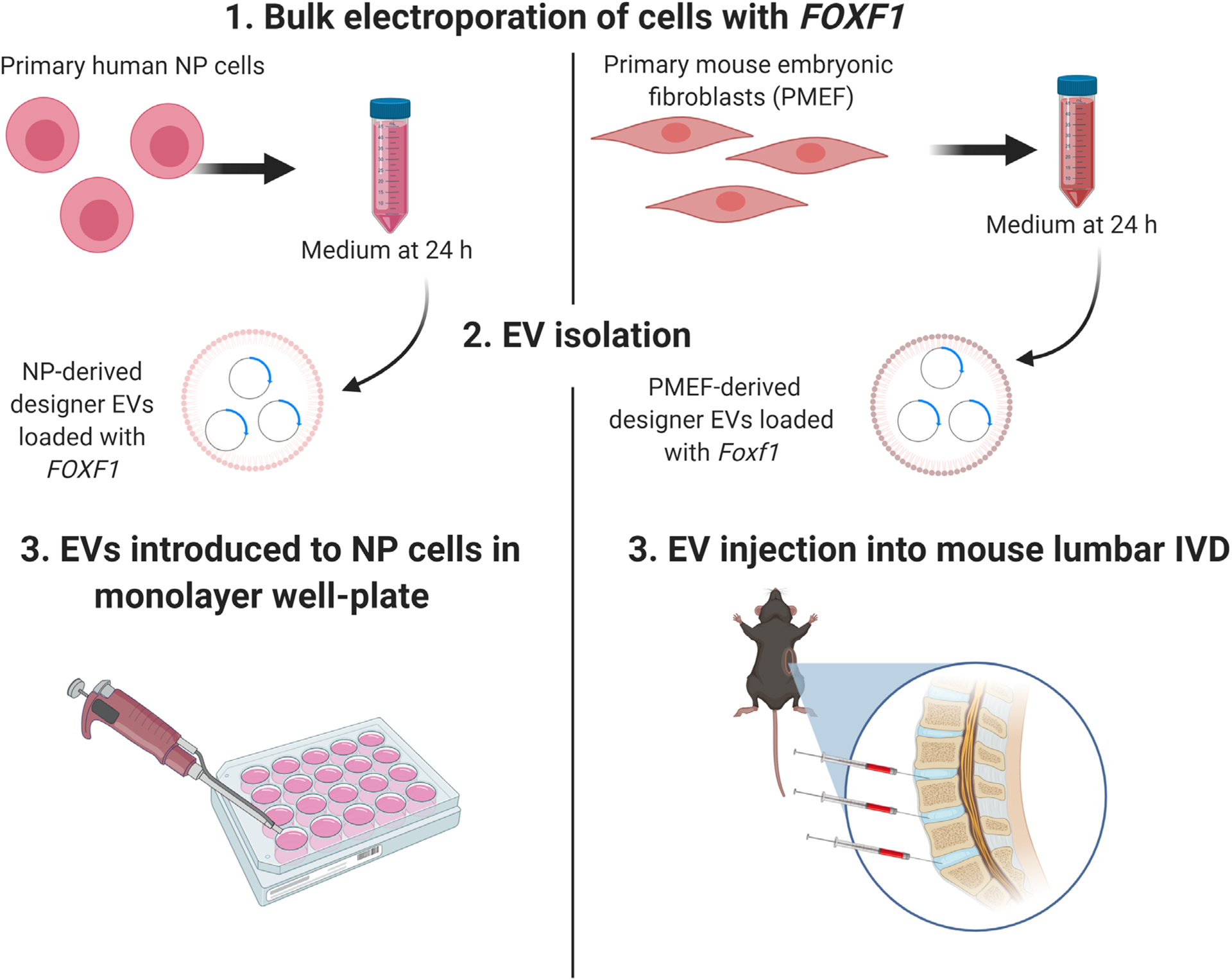
Schematic representation of delivery of FOXF1 to IVD cells *in vitro* and *in vivo* using engineered EVs. (1) First, human NP cells and mouse fibroblasts were transfected with FOXF1 respectively and medium was collected 24 h post-transfection. (2) EVs were isolated by ultracentrifugation and (3) cultured with NP cells in 24-well plates *in vitro* (human) or injected into mice L4/5, L5/6, L6/S1 discs after performing a left unilateral incision *in vivo*. Illustrations created with licensed BioRender.com software.

**Table 1.** Human autopsy and surgical specimen demographics.

Autopsy samples					Surgical samples			
ID	Sex	Age (years)	Level	Averaged grade	ID	Sex	Age (years)	Level
Hu-4*	Female	49	L2-L3	2.5	Hs-2*	Male	26	L5-S1
Hu-6*	Male	45	L2-L3	3	Hs-11*	Male	28	L5-S1
Hu-7*	Female	56	L2-L3	2.5	Hs-29*	Female	70	L5-S1
Hu-9*	Female	58	L4-L5	2.5	Hs-34*	Female	19	L5-S1
Hu-13^	Female	30	L4-L5	2.5	Hs-39*	Male	60	L5-S1
Hu-15^	Female	59	L1-L2	3				
Hu-16*^	Female	19	L1-L2	1.5				
Hu-17^	Male	39	L4-L5	3.25				

* = samples used in objective 1

^ = samples used in objective 2

**Table 2.** OriGene technologies plasmid and antibiotic details.

Name	Catalog number	Ref sequence from NIH	Species	Antibiotic resistance	Vector
*FOXF1*	RG218259	NM_001451	Human	Ampicillin (100 μg/mL)	pCMV6-AC-GFP
*Foxf1*	MR225056	NM_010426	Mouse	Kanamycin (25 μg/mL)	pCMV6-Entry
pCMV6	PS100001	NA	NA	Kanamycin (25 μg/mL)	pCMV6-Entry

**Table 3.** TaqMan gene expression primer details.

Category	Target gene	Assay ID
Control	*18S*	4333760F
Healthy NP makers	*Brachyury*	Hs00610080_m1 (human), Mm00436877_m1 (mouse)
	*FOXF1*	Hs00230962_m1(human), Mm00487497_m1(mouse)
	*KRT19*	Hs00761767_s1
	*SOX9*	Hs01107818_m1
Inflammatory cytokines	*IL-1β*	Hs00174097_m1
	*IL6*	Hs00174131_m1
	*TNFα*	Hs01113624_g1
Nerve growth	*NGF*	Hs00171458_m1
Matrix degrading enzymes	*MMP13*	Hs00233992_m1
Matrix genes	*COL2*	Hs00264051_m1
	*ACAN*	Hs00153936_m1

## List of Abbreviations

ACAN: aggrecan
AF: annulus fibrosus
CEP: cartilage end plate
CHTN: Cooperative Human Tissue Network
COL2: collagen type II
DBP: discogenic back pain
DMEM: Dulbecco’s modified Eagle’s medium
DMMB: dimethylmethylene blue
ECM: extracellular matrix
EVs: extracellular vesicles
FBS: fetal bovine serum
FOXF1: forkhead-box F1 (human)
Foxf1: forkhead-box f1 (mouse)
GAG: glycosaminoglycan
GDF: growth and differentiation factor
KRT19: keratin 19
IL: interleukin
IVD: intervertebral disc
LBP: low-back pain
MMP: matrix metalloproteinase
MSCs: mesenchymal stem cells
NGF: nerve growth factor
NP: nucleus pulposus
pCMV6: pCMV6 sham vector control
PBS: phosphate-buffered saline
PMEF: primary mouse embryonic fibroblast
P/S: penicillin/streptomycin
SOX9: SRY-box transcription factor 9
TNF-α: tumor necrosis factor alpha
